# The management of acute retroviral syndrome

**DOI:** 10.1186/1758-2652-13-S4-P2

**Published:** 2010-11-08

**Authors:** A Hristea, R Petre, S Gliga, I Olaru, M Ion, R Moroti, C Popescu, R Mihailescu, M Radulescu, V Arama

**Affiliations:** 1 National Institute for Infectious Diseases, Bucharest, Romania

## Background

Guidelines recommend starting treatment in HIV infected patients earlier than before, but there is no consensus on the exact moment when treatment should be initiated. Would treatment started and maintained in patients with acute retroviral syndrome, make an impact on disease progression, and outcome?

## Study objective

to follow and assess patients diagnosed with acute retroviral syndrome (ARS) in our facility and evaluate short-term differences between patients on continuous antiretroviral treatment and patients who were treated only during the acute phase.

## Materials and methods

A retrospective study of patients diagnosed with ARS in our facility between 1999-2009, who had previously tested negative at an ELISA test and consequently had a positive or negative ELISA and a viral load of over 10.000 copies/ml were included in the study. Patients were divided into two groups: patients who were only treated initially and then therapy was stopped (group 1) and patients who continued treatment (group 2).

## Results

Sixteen patients met the criteria (11 males and 5 females), median age of 28.5 years. Eleven were diagnosed after 2005. Patients were followed for a median duration of 42 months (12-132 months). Five patients were included in the first group and 11 in group 2. Median age of patients was higher in group 1 vs group 2 (34 vs 25 years). Symptoms and signs reported at diagnosis were: fatigue (16 patients), fever (12), dysphagy (12), lymph node enlargement (10), rash (10), myalgias (8), exudative pharyngitis (6), thrush (6), oral ulcerations (6), weight loss (6), meningitis (4) and genital ulcers (2). The median CD4 count at diagnosis was 394 cells/mm3 (range 123-1184 cells/mm3) and the median viral load was 772000 copies/ml. The total duration of antiretroviral therapy (ARVT) was between 12 and 100 months. The median CD4 count at the last evaluation was higher in group 2 (579 cells/mm3) vs group 1 (467 cells/mm3), but the rise in CD4 count during follow-up did not differ significantly between groups, since patients in group 2 had a median CD4 count on inclusion of 397 cells/mm3 vs 280 cells/mm3 in group 1. Median viral load in patients without ARVT was 6358 copies/ml while in those under therapy was undetectable.

## Conclusions

Even though the follow up period was relatively short (median of 72 months for group 1 and 27 months for group 2) continuing therapy after the acute phase did not have an impact on the immunological status of the patients.

